# Vertical Integration of Electronic Health Records in Medical Consortiums: Dynamic Modeling Approach Based on the Evolutionary Game Theory

**DOI:** 10.2196/41528

**Published:** 2023-03-06

**Authors:** Shenghu Tian, Yu Chen

**Affiliations:** 1 Business School Yunnan University of Finance and Economics Kunming China; 2 School of Management and Economics Kunming University of Science and Technology Kunming China

**Keywords:** medical consortium, EHRs, evolutionary game, system dynamics, medical information resources

## Abstract

**Background:**

China has continuously issued policies to speed up the interconnection, mutual recognition, sharing of medical information systems, and data integration management across regions and institutions. However, the vertical integration of electronic health records (EHRs) within the medical consortium is hampered by “poor mechanism and insufficient motivation” and the phenomenon of “free riding” among participating medical institutions, which makes the integration less effective.

**Objective:**

We hope to clarify the game mechanism of stakeholders in the vertical integration of EHRs, and put forward targeted policy suggestions for improvement.

**Methods:**

We constructed the “government-hospital-patient” tripartite evolutionary game model based on the detailed analysis of the research problems and their assumptions. We then simulated the game strategies and outcomes of each participant using the system dynamics approach to reveal the long-term strategy evolution mechanism of the core participants in the vertical integration of EHRs in the medical consortium, as well as the influencing factors and action mechanisms of each party’s strategy evolution to provide references for improving relevant policies.

**Results:**

The evolutionary game system could eventually reach an optimal equilibrium, but in areas where the government was required to be in a dominant position, patient supervision was necessary to have a positive role, while a reasonable reward and punishment mechanism can promote active participation of hospitals.

**Conclusions:**

The effective way to achieve the goal of vertical integration of EHRs in the medical consortium is to build a multiagent coordination mechanism under the guidance of the government. Meanwhile, it is necessary to establish a scientific integration performance evaluation mechanism, a reward and punishment mechanism, and a benefit distribution mechanism to promote the healthy development of vertical integration of EHRs in medical consortiums.

## Introduction

The construction of a medical consortium is an important institutional innovation to deepen medical reform and a specific practical path to achieve graded diagnosis and treatment, which plays a vital role in the reform and development of medical and health undertakings in China. It is generally believed that a medical consortium is a medical service community with high-level medical institutions (tertiary hospitals) as the core institution (also named the leading unit) and other medical and health institutions at various levels in the region united as participating units (also known as member units) to vertically integrate medical and health resources, thereby eliminating fragmentation of medical services and achieving integration of services, responsibilities, benefits, and management [1]. The accessibility, continuity, and radiation of medical services are enhanced, and the quality and efficiency are improved, via the integration of cross-regional and cross-level medical services, resources, technologies, and information [2]. Since the promulgation of the “Pilot Work Plan for the Construction of Urban Medical Consortium” [3], relevant policies have repeatedly emphasized strengthening the integrated management of medical consortiums, realizing the continuous recording of electronic health records (EHRs) and electronic medical records, and gradually realizing the effective sharing of medical and health information within the medical consortium, to improve the efficiency of treatment and reduce medical costs. The “14th Five-Year Plan for the Implementation of Quality and Efficient Medical and Health Services,” released in July 2021 [4], also includes accelerating the interconnection, mutual recognition, and sharing of cross-regional and cross-institutional information systems, as well as data integration and management as key projects. The construction of a medical consortium has thus become an important strategic measure to build an integrated medical and health service system in China.

EHRs refer to the systematic digital record of health information, such as disease treatment, immunization, chronic disease management, genetic history, throughout the life cycle of individual citizens through a variety of collection channels. The existing EHRs are scattered in different medical and health institutions at all levels, which seriously restricts the healthy and orderly development of a medical consortium. The vertical integration of EHRs refers to the collection, sorting, and ordering of EHRs among medical institutions at different levels within a medical consortium [5]. The purpose of EHRs is to break the decentralized pattern, promote the collaboration and hierarchical diagnosis and treatment within the medical consortium, promote the in-depth cooperation and organizational collaboration within the medical consortium, and help the development of the medical consortium from loose to compact. The integration of medical information resources has always been the core of medical and health information research. Although existing studies focused on the integration of diagnosis and treatment information resources within medical institutions, attention given to the integration and sharing of EHRs across institutions is far from adequate. The reasons for this are as follows: (1) the policies related to the integration of medical consortium and EHRs have not been proposed for a long time, and the policy effect has not been fully manifested; and (2) the shackles of “poor mechanism and insufficient motivation” are deeply rooted in the integration of medical resources across institutions, and the phenomenon of “free riding” by participating medical institutions is prominent, which makes the integration ineffective.

The game theory is suitable for the study of multiagent behavior. Evolutionary game is favored because its bounded rationality assumption of game players is more realistic, which is widely used in various fields, such as multiple governance, organizational coordination, and information behavior. Cross-institutional information resource sharing often involves many stakeholders, and it could gradually approach the evolutionary stable state after repeated games. Some studies have tried to use the evolutionary game method to perform relevant research in the medical and health field, such as studying doctor-patient disputes under government regulation [6], evolutionary game of a 2-way referral mechanism [7], emergency management of public emergencies [8], knowledge sharing in online health communities [9], drug safety and public health quality supervision [10,11], and integrated health care systems [12]. Some studies also use evolutionary game and simulation methods to explore the willingness of medical data sharing, but these only consider the 2 parties of patients and medical service institutions [13], and fail to consider the willingness and influence mechanism of cross-agency data sharing of the government and other multiparties under the organization mode of the medical consortium. The government, hospitals, and patients are the 3 direct stakeholders involved in the vertical integration of EHRs. To make all parties act in a unified manner in order to achieve the collective goal of resource integration, it is necessary to understand the long-term game strategy and its evolutionary stability of all parties. In addition, most previous studies used qualitative analysis methods such as speculative reasoning, expert interviews, or text analysis, and few studies used mathematical methods to discuss the issues shared by EHRs across institutions.

The essence of cross-organizational EHRs integration within a medical consortium is not a technical implementation but an organizational synergy problem, the core of which is the progressive evolutionary game problem of multiple stakeholders. In particular, participating medical institutions and patients have more evolutionary characteristics of progressive learning, and government behavior affects the development direction of EHRs integration. In this paper, we used evolutionary game analysis to incorporate the government, hospitals, and patients into a system model to explore the long-term strategy evolution of the 3 parties in the vertical integration of cross-organizational EHRs within a medical consortium, as well as the influencing factors and mechanisms of each party’s strategy evolution. Finally, we proposed corresponding countermeasures based on data simulation using system dynamics. The findings of the study can provide a reference basis for the smooth implementation of vertical integration of EHRs within a medical consortium.

## Methods

### Construction of an Evolutionary Game Model

#### Problem Statements and Assumptions

From the perspective of stakeholders, the core participants involved in the vertical integration of EHRs are mainly the government, hospitals, and patients. As the main driving force of the hierarchical diagnosis and treatment system, the government has made every effort to promote the vertical integration of EHRs in the medical consortium through top-level design and the introduction of relevant policies to optimize the allocation of medical resources. However, local governments may make every effort to promote it or treat it negatively, due to the impact of protectionism, high costs, human resources, and complexity of implementation. Therefore, the behavior strategies of the local government are either “trying to promote it” or “treating it negatively.”

#### Hypothesis 1

For local governments, if they try their best to promote the integration of EHRs, the income, such as social value, public praise, superior rewards, will be *R_g_* and the cost will be *C_g_*; when the local government takes a negative attitude, it will neither give subsidies or rewards to the hospitals and patients nor impose penalties. The cost is *K_g_* (*C_g_*>*K_g_*) in such cases, and the local government will be punished as *F_g_* (including the accountability of the superior government, government credibility, and reputation loss). When the hospital actively participates in the information integration under the policy effect and achieves good results in practice, the boundary between the local government’s “efforts to promote” and “negative treatment” will become very vague. Therefore, the local government will not be held accountable by the superior government, nor will there be potential punishment such as loss of credibility and reputation.

As the owner of EHRs, hospitals’ participation attitude directly affects the success or failure of the vertical integration of EHRs. Existing research shows that the leading large hospitals worry about weakening the absolute dominant position of medical resources, while the small hospitals participating in the medical consortium may negatively treat the integration of information resources to maintain their own economic interests and social reputation [14]. However, both the leading hospitals and member units may actively respond to national policies, abandon “free riding” opportunism, and actively participate in the vertical integration project of EHRs. Therefore, the behavior strategies of the hospital are active participation or passive participation.

#### Hypothesis 2

For the hospital, the revenue under normal operation is *R_h_*. If the hospital actively participates in the sharing and utilization of cross-institutional EHRs, the reward subsidy from the superior government is *F_h_*, and the cost is *C_h_*. If the hospital chooses a negative strategy, its cost is *K_h_* (*C_h_*>*K_h_*), and the resulting damage to the overall social interests is *R_p_*, which will be borne by the public (especially patients). When patients find that their EHRs cannot be shared and utilized across institutions, if they complain to the superior supervision organization, the hospital will be punished as *M_h_*. If the patients do not choose to complain, no supervision cost will be incurred.

As the biggest beneficiary of the hierarchical diagnosis and treatment system, the patients have the right and obligation to supervise and complain about the interconnection and availability of EHRs in the medical consortium. The patients may supervise hospital behaviors to protect their own rights and interests and complain to the medical and health supervision department, but they may also give up their right to supervision because of negative ideas such as “It’s better to save trouble” and “supervision is the same as non-supervision” or because they think that the supervision cost is too high. Therefore, the behavior strategy space of patients is participating in supervision or giving up supervision.

#### Hypothesis 3

For patients, the cost of active participation in supervision is *C_p_*, and the reward from the government is *F_p_*; if patients give up supervision, they will neither incur costs nor receive government incentives.

The behaviors of local governments, hospitals, and patients all conform to the bounded rationality hypothesis of the evolutionary game theory. In the game process, each party continuously adjusts its own strategies through the behaviors of the other 2 parties, and finally reaches a stable state after repeated games.

#### Construction of a Tripartite Profit Matrix

Assuming that the ratio of local government choosing to promote is *x*, the ratio of its negative treatment is 1 − *x*. If the rate of active participation is *y*, the rate of passive participation is 1 − *y*. If the rate of patients choosing supervision complaints is *z*, the rate of giving up supervision is 1 − *z*, and *x*, *y*, *z* ∈ [0, 1]. The relevant parameter settings and their meanings are shown as Table 1.

According to the aforementioned analysis and assumptions, the game payoff matrix of the 3 core players in the vertical integration of EHRs in the medical consortium under different strategic combinations is shown in Table 2.

**Table 1:** Relevant parameters setting and their meanings.

Parameters	Meanings
*R*_g_	Benefits when the local government tries to promote EHR^a^ integration
*C*_g_	Costs when the local government pushes hard
*K*_g_	Costs when the local government treats EHR integration negatively
*F*_g_	Punishment received when the local government treats EHR integration negatively
*R*_h_	Revenue when the hospital is in normal operation
*C*_h_	Costs when hospitals are actively involved in EHR integration
*K*_h_	Costs when hospitals are passively involved in EHR integration
*F*_h_	Government subsidies when hospitals actively participate in EHR integration
*M*_h_	Punishment when the hospital participates passively in EHR integration
*F*_p_	Rewards received when patients actively participate in supervision
*C*_p_	Costs when patients actively participate in supervision
*R*_p_	Social damage caused by negative hospital participation

^a^EHR: electronic health record.

**Table 2:** Evolutionary game payoff matrix of the local government, hospital, and patient.

Game players				Local governments			
				Push hard (*x*)		Negative treatment (1 − *x*)	
**Hospital**							
	**Actively participation (*y*)**						
		Patient					
			Participation in supervision (*z*)	*R*_g_ − *C*_g_ − *F*_h_ − *F*_p_, *R*_h_ − *C*_h_ + *F*_h_, *F*_p_ − *C*_p_		−*K*_g_*, R*_h_ − *C*_h_, −*C*_p_	
			Waiver of supervision (1 − *z*)	*R*_g_ − *C*_g_ − *F*_h_, *R*_h_ − *C*_h_ + *F*_h_, 0		−*K*_g_*, R*_h_ − *C*_h_, 0	
	**Passive participation (1 − *y*)**						
		Patient					
			Participation in supervision (*z*)	*R*_g_ − *C*_g_ + *M*_h_, −*K*_h_ − *M*_h_, *F*_p_ − *C*_p_ − *R*_p_		−*K*_g_ − *F*_g_, −*K*_h_, −*C*_p_	
			Waiver of supervision (1 − *z*)	*R*_g_ − *C*_g_ + *M*_h_, −*K*_h_ − *M*_h_, −*R*_p_		−*K*_g_ − *F*_g_, −*K*_h_, −*R*_p_	

### Behavior Equilibrium Analysis of Evolutionary Game

#### Expected Profit of Game Players

Let *E_g_*_1_ be the expected return when the government pushes hard, *E_g_*_2_ be the expected return when the government treats EHR integration negatively, and *E_g_* be the average return under the 2 strategies of the government pushing hard and treating negatively, then:

*E_g_* = *xE_g_*_1_ + (1 − *x*)*E_g_*_2_
**(1)**

*E_g_*_1_ = *yz*(*R_g_* − *C_g_* − *F_h_* − *F_g_*) + *y*(1 − *z*)(*R_g_* − *C_g_* − *F_h_*) + *z*(1 − *y*)(*R_g_* − *C_g_* + *M_h_*) + (1 − *y*)(1 − *z*)(*R_g_* − *C_g_* + *M_h_*) **(2)**

*E_g_*_2_ = *yz*(−*K_g_*) + *y*(1 − *z*)(−*K_g_*) + *z*(1 − *y*)(−*K_g_* − *F_g_*) + (1 − *y*)(1 − *z*)( −*K_g_* − *F_g_*) **(3)**

Let *E_h_*_1_ be the expected benefit when the hospital actively participates, *E_h_*_2_ be the expected benefit when the hospital passively participates, and *E_h_* be the average benefit in both cases of active and passive hospital participation, then we have:

*E_h_* = *yE_h_*_1_ + (1 − *y*)*E_h_*_2+_
**(4)**

*E_h_*_1_ = *xz*(*R_h_* − *C_h_* + *F_h_*) + *x*(1 − *z*)(*R_h_* − *C_h_* + *F_h_*) + *z*(1 − *x*)(*R_h_* − *C_h_*) + (1 − *x*)(1 − *z*)(*R_h_* − *C_h_*) **(5)**

*E_h_*_2_ = *xz*(−*K_h_* − *M_h_*) + *x*(1 − *z*)(−*K_h_* − *M_h_*) + *z*(1 − *x*)(−*K_h_*) + *z*(1 − *x*)(−*K_h_*) + (1 − *x*)(1 − *z*)(−*K_h_*) **(6)**

Let *E_p_*_1_ be the expected benefit when the hospital actively participates, *E_p_*_2_ be the expected benefit when the hospital passively participates, and *E_p_* be the average benefit in both cases of active and passive hospital participation, then we have:

*E_p_* = *zE_p_*_1_ + (1 − *z*)*E_p_*_2_
**(7)**

*E_p_*_1_ = *xy*(*F_P_* − *C_P_*) + *y*(1 − *x*)(−*C_P_*) + *x*(1 − *y*)(*F_P_* − *C_P_* − *R_P_*) + (1 − *x*)(1 − *y*)(−*C_P_*) **(8)**

*E_p_*_2_ = *x*(1 − *y*)(−*R_p_*) + (1 − *x*)(1 − *y*)(−*R_p_*) **(9)**

#### Replication Dynamic Equation of Game Players

Replication dynamic equations can not only reflect the speed of evolution, but also determine the direction of decision evolution of game players. According to the calculation method proposed by Friedman [15], we constructed the replication dynamic equation under the active state of each game player.

Combining equations 1-3, we could construct the replication dynamic equation when the government tries to promote EHR integration as follows:

*F*(*x*) = *dx*/*dt* = *x*(*E_g_*_1_ − *E_g_*) = *x*(1 − *x*)(*Eg*_1_ − *E_g_*_2_)

= *x*(1-*x*){[*yz*(*R_g_* − *C_g_* − *F_h_* − *F_p_*) + *y*(1 − *z*)(*R_g_* − *C_g_* − *F_h_*) + *z*(1 − *y*)(*R_g_* − *C_g_* + *M_h_*) + (1 − *y*)(1 − *z*)(*R_g_* − *C_g_* + *M_h_*)] − [*yz*(−*K_g_*) + *y*(1 − *z*)(−*K_g_*) + *z*(1 − *y*)(−*K_g_* − *F_g_*) + (1 − *y*)(1 − *z*)(−*K_g_* − *F_g_*)]}

= *x*(1 − *x*)[(*R_g_* + F*_g_* + *M_h_* + *K_g_* − *C_g_*) − *y*(*zF_p_* + *F_g_* + *F_h_* + *M_h_*)] **(10)**

Combining equations 4-6, the replication dynamic equation for active hospital participation could be constructed as follows:

*F*(*y*) = *dy*/*dt* = *y*(*Eh*_1_ − *E_h_*) = *y*(1 − *y*)(*Eh*_1_ − *Eh*_2_)

= *y*(1 − *y*){[*xz*(*R_h_* − *C_h_* + *F_h_*) + *x*(1 − *z*)(*R_h_* − *C_h_* + *F_h_*) + *z*(1 − *x*)(*R_h_* − *C_h_*) + (1 − *x*)(1 − *z*)(*R_h_* − *C_h_*)] − [*xz*(−*K_h_* − *M_h_*) + *x*(1 − *z*)(−*K_h_* − *M_h_*) + *z*(1 − *x*)(−*K_h_*) + (1 − *x*)(1 − *z*)(−*K_h_*)]}

= *y*(1 − *y*)[(*R_h_* + *K_h_* − *C_h_*) − *x*(*F_h_* + *M_h_*)] **(11)**

Similarly, combining equations 7-9, the replication dynamic equation could be constructed when the patient is actively supervised as follows:

*F*(*z*) = *dz*/*dt* = *z*(*Ep*_1_ − *E_p_*) = *z*(1 − *z*)(*Ep*_1_ − *Ep*_2_)

= *z*(1 − *z*){[*xy*(*F_p_* − *C_p_*) + *y*(1 − *x*)(−*C_p_*) + *x*(1 − *y*)(*F_p_* − *C_p_* − *R_p_*) + (1 − *x*)(1 − *y*)(−*C_p_*)] − [*x*(1 − *y*)(−*R_p_*) + (1 − *x*)(1 − *y*)(−*R_p_*)]}

= *z*(1 − *z*)[*x*(*F_p_* − *R_p_* − *yR_p_*)+(1 − *y*)*R_p_* − *C_p_*] **(12)**

#### Equilibrium Analysis of the Evolutionary Game System

Combining equations 10-12, we can get the 3D dynamic system of the game participants (ie, the government, hospitals, and patients), which is given as follows:

*F*(*x*) = *dx*/*dt* = *x*(1 − *x*)[(*R_g_* + *F_g_* + *M_h_* + *K_g_* + *C_g_*) − *y*(*zF_p_* + *F_g_* + *F_h_* + *M_h_*)]

*F*(*y*) = *dy*/*dy* = *y*(1 − *y*)[(*R_h_* + *K_h_* − *C_h_*) + *x*(*F_h_* + *M_h_*)]

*F*(*z*) = *dz*/*dt* = *z*(1 − *z*)[*x*(*F_p_* − *R_p_* + *yR_p_*) + (1 − *y*)*R_p_* − *C_p_*] **(13)**

By making the 3 replicated dynamic equations in equation (13) equal to 0, we can obtain 8 pure strategies Nash equilibrium solutions of the evolutionary game system, namely, (0, 0, 0), (0, 0, 1), (0, 1, 0), (0, 1, 1), (1, 0, 0), (1, 0, 1), (1, 1, 0), and (1, 1, 1). Existing studies in the field have proved that only the stability of the aforementioned 8 local equilibrium points can be considered in the analysis of a 3-party evolutionary game [16], and that it is not necessary to consider the Nash equilibrium solution *E* (x*, y*, z*) of the mixed strategy. The sign of Jacobian matrix can be used to judge whether the aforesaid 8 local equilibrium points are the evolutionary stability strategy of the system, and the condition is det(*J*)>0 and tr(*J*)<0. The Jacobian matrix can be obtained by solving the partial derivatives of the 3 equations in equation (13) with respect to *x*, *y* and *z*, respectively:


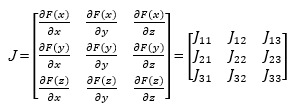


*J*_11_ = (1 − 2*x*)[(*R_g_* + *F_g_* + *M_h_* + *K_g_* − *C_g_*) − *y*(*zF_p_* + *F_g_* + *F_h_* + *M_h_*)];

*J*_12_ = −*x*(1 − *x*)(*zF_p_* + *F_g_* + *F_h_* + *M_h_*);

*J*_13_ = −*xy*(1 − *x*)*F_p_*;

*J*_21_ = *y*(1 − *y*)(*F_h_* + *M_h_*);

*J*_22_ = (1 − 2*y*)[(*R_h_* + *K_h_* − *C_h_*) + *x*(*F_h_* + *M_h_*)];

*J*_23_ = 0;

*J*_31_ = z(1 − *z*)(*F_p_* − *R_p_* + *yR_p_*);

*J*_32_ = −*z*(1 − *z*)(1 − *x*)*R_p_*;

*J*_33_ = (1 − 2*z*)[*x*(*F_p_* − *R_p_* + *yR_p_*) + (1 − *y*)*R_p_* − *C_p_*];

det(*J*) *=*
*J*_11_*J*_22_*J*_33_
*+ J*_12_*J*_23_*J*_31_
*+ J*_13_*J*_21_*J*_32_ − *J*_13_*J*_22_*J*_31_ − *J*_12_*J*_21_*J*_33_ − *J*_11_*J*_23_*J*_32_
*= xzy*^2^(1 − *x*)^2^(1 − *y*)(1 − *z*) (*F_h_ + M_p_*)*F_p_R_p_ + xyz*(1 − *x*)(1 − 2*y*)(1 − *z*)(*F_p_ + yR_p_* − *R_p_*)[−*C_h_ + K_h_ + R_h_ + x*(*F_h_ + M_h_*)]*F_p_ + xy*(1 − *x*)(1 − *y*)(1 − 2*z*)(*F_h_ + M_h_*)[(−*C_p_ +* (1 − *y*)*R_p_ + x*(*F_p_ + yR_p_* − *R_p_*)](*F_g_ + F_h_ + zF_p_ + M_h_*) *+* (1 − 2*x*)(1 − 2*y*)(1 − 2*z*)[−*C_p_ +* (1 − *y*)*R_p_ + x*(*F_p_ + yR_p_* − *R_p_*)][−*C_h_ + K_h_ + R_h_ + x*(*F_h_ + M_h_*)][−*C_g_ + F_g_ + K_g_ + M_h_ + R_g_* − *y*(*F_g_ + F_h_ + zF_p_ + M_h_*)] **(14)**

tr(*J*) = *J*_11_ + *J*_22_ + *J*_33_ = (1 − 2*x*)[−*C_g_* + *F_g_* + *K_g_* + *M_h_* + *R_g_* − *y*(*F_g_* + *F_h_* + *zF_p_* + *M_h_*)] + (1 − 2*y*)[−*C_h_* + *K_h_* + *R_h_* + *x*(*F_h_* + *M_h_*)] + (1 − 2*z*)[−*C_p_* + (1 − *y*)*R_p_* + *x*(*F_p_* + *yR_p_* − *R_p_*)] **(15)**

The det(*J*) and tr(*J*) values of 8 local equilibrium points can be calculated from equations 14 and 15 and judgment conditions, as shown in Table 3.

So far, the evolutionary game system has achieved the stable state under certain conditions. However, it can be seen from Table 3 that the det(*J*) and tr(*J*) values are determined by the values of many parameters. The existing conditions and calculation methods make it difficult to determine whether the aforesaid 8 equilibrium points are the evolutionary stability strategy, which indicates that it is not clear whether there are equilibrium points in the evolutionary game system. System dynamics can analyze the complex dynamic evolution process of evolutionary game models under the conditions of bounded rationality and asymmetric information [17], and can explore and reveal the potential mechanism of problems and seek key strategies to solve problems [18]. Therefore, we further use the system dynamics tool to simulate the impact of uncertainty factors on the stability of the evolutionary game system, so as to provide decision-making reference for formulating relevant policies and optimizing the dynamic mechanism of EHRs integration.

**Table 3:** The det(*J*) and tr(*J*) of the Jacobian matrix.

Equilibrium point	Det(*J*)	Tr(*J*)
(0, 0, 0)	(*R*_p_ − *C*_p_)(*K*_h_ + *R*_h_ − *C*_h_)(*F*_g_ + *K*_g_ + *M*_h_ + *R*_g_ − *C*_g_)	*F*_g_ + *K*_g_ + *K*_h_ + *M*_h_ + *R*_g_ + *R*_h_ + *R*_p_ − *C*_g_ − *C*_h_ − *C*_p_
(0, 0, 1)	(*C*_p_ − *R*_p_)(*K*_h_ + *R*_h_ − *C*_h_)(*F*_g_ + *K*_g_ + *M*_h_ + *R*_g_ − *C*_g_)	*C*_p_ + *F*_g_ + *K*_g_ + *K*_h_ + *M*_h_ + *R*_g_ + *R*_h_ − *R*_p_ − *C*_g_ − *C*_h_
(0, 1, 0)	−*C*_p_(*C*_h_ − *K*_h_ − *R*_h_)(*K*_g_ + *R*_g_ + *C*_g_ − *F*_h_)	*C*_h_ − *C*_p_ − *F*_h_ + *K*_g_ − *K*_h_ + *R*_g_ − *R*_h_ − *C*_g_
(0, 1, 1)	*C*_p_(*C*_h_ − *K*_h_ − *R*_h_)(*K*_g_ + *R*_g_ − *C*_g_ − *F*_h_ − *F*_p_)	*C*_h_ + *C*_p_ − *F*_h_ − *F*_p_ + *K*_g_ − *K*_h_ + *R*_g_ − *R*_h_ − *C*_g_
(1, 0, 0)	(*F*_p_ − *C*_p_)(*C*_g_ − *F*_g_ − *K*_g_ − *M*_h_ − *R*_g_)(*F*_h_ + *K*_h_ + *M*_h_ + *R*_h_ − *C*_h_)	*C*_g_ − *C*_h_ − *C*_p_ − *F*_g_ + *F*_h_ + *F*_p_ − *K*_g_ + *K*_h_ − *R*_g_ + *R*_h_
(1, 0, 1)	(*C*_p_ − *F*_p_)(*C*_g_ − *F*_g_ − *K*_g_ − *M*_h_ − *R*_g_)(*F*_h_ + *K*_h_ + *M*_h_ + *R*_h_ − *C*_h_)	*C*_g_ − *C*_h_ + *C*_p_ − *F*_g_ + *F*_h_ − *F*_p_ − *K*_g_ + *K*_h_ − *R*_g_ + *R*_h_
(1, 1, 0)	(*F*_p_ − *C*_p_)(*C*_g_ + *F*_h_ − *K*_g_ − *R*_g_)(*C*_h_ − *F*_h_ − *K*_h_ − *M*_h_ − *R*_h_)	*C*_g_ + *C*_h_ − *C*_p_ + *F*_p_ − *K*_g_ − *K*_h_ − *M*_h_ − *R*_g_ − *R*_h_
(1, 1, 1)	(*C*_p_ − *F*_p_)(*C*_g_ − *F*_h_ + *F*_p_ − *K*_g_ − *R*_g_)(*C*_h_ − *F*_h_ − *K*_h_ − *M*_h_ − *R*_h_)	*C*_g_ + *C*_h_ + *C*_p_ − *K*_g_ + *K*_h_ − *M*_h_ − *R*_g_ − *R*_h_

### Construction of the System Dynamics Simulation Model

**Figure 1:**
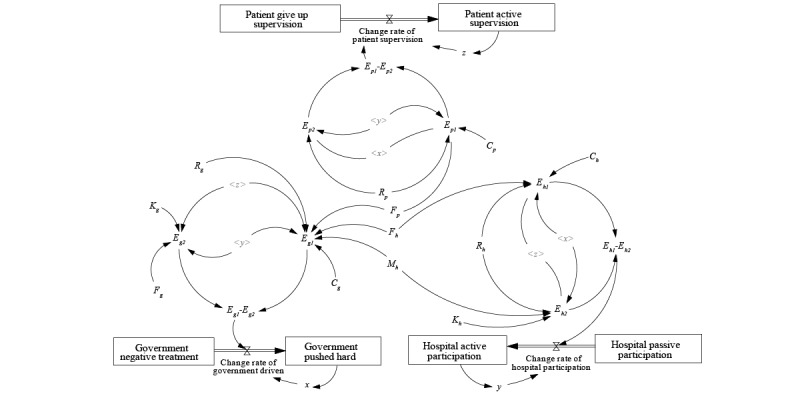
The system dynamics simulation model of the 3-party evolutionary game.

According to the aforementioned analysis, we used Vensim PLE (Ventana Systems, Inc) to build a system dynamics model of the evolutionary game among the government, hospitals, and patients in the vertical integration of EHRs in the medical consortium, as shown in Figure 1. In the figure, *x*, *y*, and *z* are 3 stocks, representing the time integration of 3 rate variables, namely, the change rate of government promotion, the change rate of hospital participation, and the change rate of patient supervision, expressed by their respective replication dynamic equations; *E_g_*_1_, *E_g_*_2_, *E_h_*_1_, *E_h_*_2_, *E_p_*_1_, and *E_p_*_2_ are 6 intermediate variables, which respectively represent the expected return of each entity under different strategic states. The remaining 12 parameters are external factors outside the system boundary that may affect stock changes. The arrow lines indicate the causal relationship between 2 elements. The interaction of cause and effect acts as a causal feedback loop.

The purpose of system dynamics simulation is to reveal the law of development and change of things. The model construction focuses on the consistency, adaptability, and effectiveness of its overall structure. Therefore, the design of a system dynamics model does not require the authenticity and accuracy of parameter setting [19]. In this paper, common assignment methods are used for reference in parameter setting, and finally determined according to the experience of the interviewed experts. The specific parameter settings are as follows: initial time=0 years, final time=20 years, time step=0.125 years; *R_g_*=30; *C_g_*=6; *K_g_*=3; *F_g_*=4; *R_h_*=50; *C_h_*=7; *K_h_*=5; *F_h_*=8; *M_h_*=10; *C_p_*=1; *F_p_*=2; and *R_p_*=6. It should be noted that different initial values of each parameter will not change the simulation conclusion [20].

### Ethical Considerations

Because no specific people or animals are involved in the research and no individual privacy is involved, academic ethics review is not required.

## Results

### Holistic Simulation Analysis of the Evolutionary Game Model

**Figure 2:**
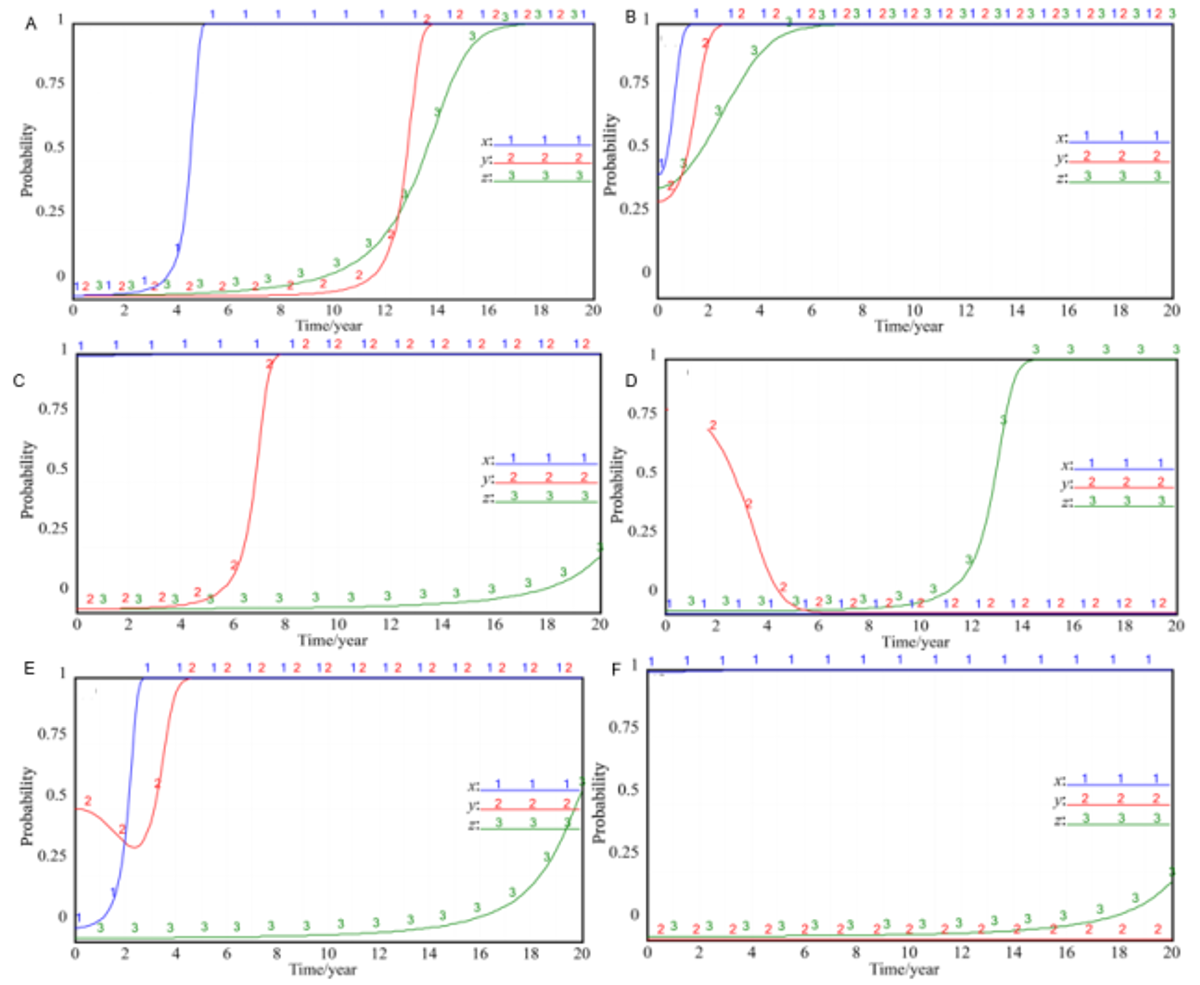
Process of the evolutionary game in the case of combined values of *x*, *y*, and *z*. (A) *x*=0.01, *y*=0.01, *z*=0.01; (B) *x*=0.45, *y*=0.35, *z*=0.4; (C) *x*=0.99, *y*=0.01, *z*=0.01; (D) *x*=0, *y*=0.8, *z*=0.01; (E) *x*=0.05, *y*=0.5, *z*=0.01; (F) *x*=0.99, *y*=0, *z*=0.01.

The initial state of the government, the hospital, and the patient in the evolutionary game is pure strategy, that is, each party’s strategy choice is 0 or 1, but the 8 possible equilibrium states formed by this are unstable. As long as one party’s strategy is slightly adjusted, the other parties will learn to imitate and then choose strategies with higher returns, and the whole game system will evolve into a new equilibrium state. Taking the (0, 0, 0) combination strategy as an example, the evolutionary game process under different values of *x*, *y*, and *z* is shown in Figure 2 (the ordinates in the figure represent the probability of strategy selection of the government, hospitals, and patients in the corresponding state).

Comparing Figure 2A-C, we can see that as long as the government’s strategy evolves in the direction of vigorous promotion, no matter how much the initial probability of active participation of hospitals and patients is, the strategy of active participation and supervision will be adopted eventually, that is, as long as X evolves from 0 to 1, the whole system will develop into a stable state of (1, 1, 1), and it shows that the evolution speed of the government’s strategy is faster than that of the hospital’s strategy. The evolution speed of the hospital’s strategy is faster than that of the patient’s strategy. At the same time, when the government’s strategy changes from 0 to 0.01 or from 1 to 0.99, it will eventually evolve into the strategy of trying to promote, showing that the strategy of trying to promote is always the best strategy of the government. During the vertical integration of cross-agency EHRs in the medical consortium, the government has always been and should be in the leading position. In specific practice, the government should take the initiative to promote rather than completely transfer the responsibility to the hospital.

When the government chooses to promote the strategy, the hospital will always reach the status of 1 before the patients. However, comparing Figure 2D,E, it can be found that when the government chooses the negative treatment strategy, even if the hospital has high enthusiasm to participate in the vertical integration of EHRs at the beginning, it will gradually choose the negative participation strategy as time goes on; however, when the hospital completely chooses the passive participation strategy, even if the government adopts the promotion strategy again, it will be difficult to promote the hospital from the passive strategy to the active participation in a short time. The enlightenment from this conclusion is that the local government should do a good job in the overall design and development planning at the beginning, or it will be difficult to achieve the national goal of vertical integration of EHRs.

It can be seen in Figure 2 that the patients were the slowest to choose the active supervision strategy, but this is always the best strategy for patients. At the same time, it was also found that once patients choose the active supervision strategy, no matter how the strategies of the government and hospital change, the patients will tend to the active supervision strategy. Through the overall simulation analysis, it can be seen that the evolutionary game system will finally reach the equilibrium state at (1, 1, 1) after the 3 parties’ strategy mutation, learning imitation, and strategy adjustment in the game process.

### Factors Influencing the Government’s Strategy Selection

**Figure 3:**
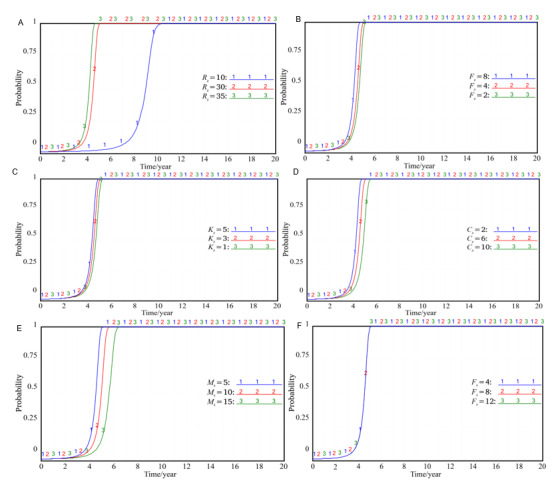
Impact of external variables on government's strategy selection. (A) The impact of *R_g_* on the choice of government's strategy. (B) The impact of *F_g_* on the choice of government's strategy. (C) The influence of *K_g_* on the choice of government's strategy. (D) The influence of *C_g_* on the choice of government's strategy; (E) The impact of *M_h_* on the choice of government's strategy; (F) The impact of *F_h_* on the choice of government's strategy.

According to the model simulation, 7 external variables such as the revenue *R_g_* and cost *C_g_* when the local government tries to promote, the cost *K_g_* and punishment *F_g_* when the local government treats the hospital negatively, the government subsidy *F_h_* to the hospital, the supervision reward *F_p_* to the patients, and the punishment *M_h_* when the local government participates in the hospital negatively will affect the local government’s strategy choice (Figure 3). It can be seen from Figure 3A-C that *R_g_*, *F_g_*, and *K_g_* have similar effects on the government’s strategy selection. In the initial value state, increasing the values of the 3 parameters will make the government’s promotion probability *x* reach 1 earlier, which means increasing the revenue when the government is trying to promote. By contrast, increasing the cost and punishment of the government’s negative treatment of the integration of EHRs will enhance the government’s enthusiasm for trying to promote. Comparatively, income has a greater impact on the choice of the government’s strategy. Comparing Figure 3D,E, it can be seen that *C_g_* and *M_h_* have similar effects on the government’s strategy. Increasing the values of the 2 parameters respectively will prolong the period for the government to choose the strategy of trying to promote, which indicates that the higher the promotion cost, the lower the probability of the government trying to promote. It is worth mentioning that when the punishment *M_h_* for passive participation in the hospital is increased to a certain extent, the government will slow down its efforts to promote the stability of the strategy, which indicates that the government cannot blindly increase its revenue by increasing the punishment. It also means that the government revenue *R_g_* pays more attention to the social value after the integration of EHRs rather than punishing the hospital. The change of *F_h_* and *F_p_* has no obvious impact on the government’s strategy. The curve trend after the change of the 2 parameters is basically the same, as shown in Figure 3E, which means that as long as the goal of integrating medical resources to improve medical efficiency can be achieved, the local government is likely to be willing to grant subsidies and supervise incentives. In other words, whether to give subsidies to hospitals or reward patients for supervision is not the main factor affecting the government’s choice of strategies.

### Factors Influencing the Hospital’s Strategy Selection

**Figure 4:**
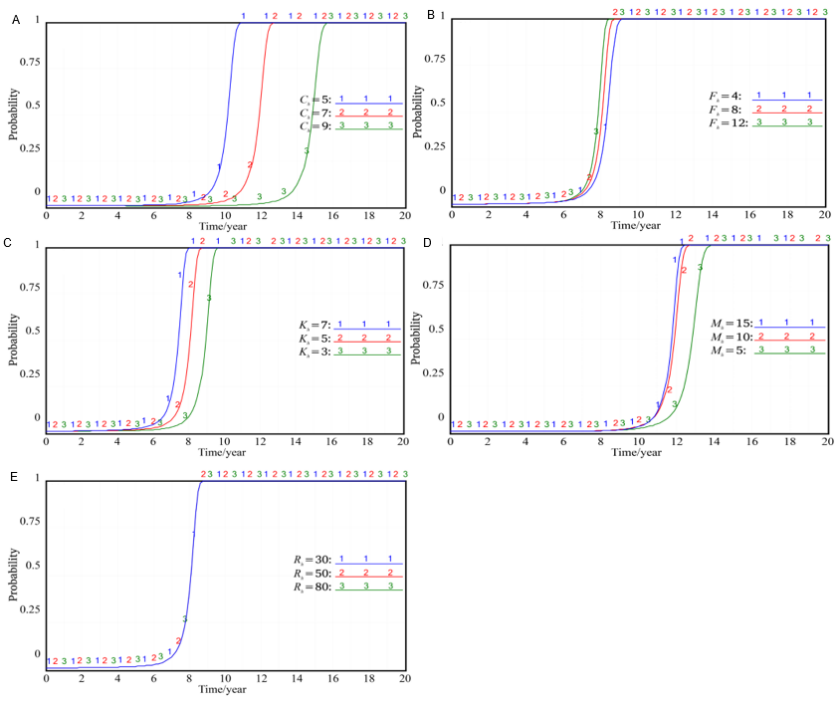
Impact of external variables on the hospital's strategy selection. (A) Impact of *C_h_* on the hospital's strategy selection. (B) Impact of *F_h_* on the hospital's strategy selection. (C) Impact of *K_h_* on the hospital's strategy selection. (D) Impact of *M_h_* on the hospital's strategy selection. (E) Impact of *R_h_* on the hospital's strategy selection.

Through simulation, it can be found that 4 external variables, namely, the cost *C_h_* when the hospital actively participates, the cost *K_h_* when the hospital passively participates, the government subsidy *F_h_* when the hospital actively participates, and the punishment *M_h_* when the hospital passively participates, will affect the choice of the enterprise strategy (Figure 4). When the value of *C_h_* is smaller or the values of *K_h_*, *F_h_*, and *M_h_* are larger, the probability of hospitals choosing the active participation strategy will be greatly increased. According to the analysis in Figure 4D, when the punishment *M_h_* for the government’s passive participation in the hospital increases to a certain extent, the marginal utility of the punishment measures will be greatly weakened, that is, when the punishment is increased to a certain extent, the incentive effect of the punishment itself will be weakened. A consistent conclusion has been reached in the analysis of the influencing factors of the government’s strategy choice.

It was also found in the simulation that the hospital’s revenue *R_h_* under normal conditions will not affect its strategy choice (Figure 4E). This shows that neither large hospitals that occupy an absolute dominant position in medical information nor small hospitals with limited resources will refuse the vertical integration of EHRs because of the amount of their own information resources. In other words, both large hospitals and small hospitals have the potential willingness to act collectively to achieve the vertical integration of EHRs. This finding is of great significance for the government to vigorously promote the vertical integration of EHRs (eg, electronic medical records).

### Factors Influencing the Patient’s Strategy Selection

**Figure 5:**
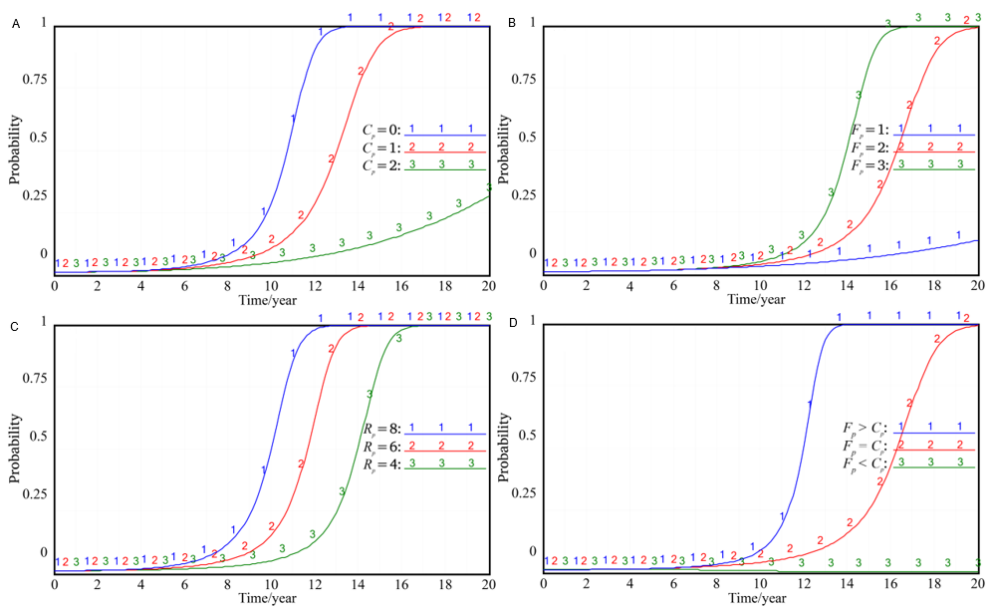
Impact of external variables on the patient's strategy selection. (A) Effect of *C_p_* on the patient's strategy selection; (B) Effect of *F_p_* on the patient's strategy selection; (C) Joint effect of *F_p_* and *C_p_* on the patient's strategy. (D) Impact of *R_p_* on the patient's strategy selection.

The factors that affect the patient’s strategy selection mainly include the cost *C_p_* and the reward *F_p_* when the patient actively participates in the supervision, as well as the social damage *R_p_* caused by the hospital’s negative participation (Figure 5). We can see in Figure 5A,B that when the patient’s supervision cost *C_p_* becomes larger or the government reward *F_p_* becomes smaller, the probability of patients choosing the active supervision strategy will obviously decrease. It can be seen from the simulation that the probability of patients choosing the active supervision strategy is determined by the difference between *F_p_* and *C_p_*, as shown in Figure 5C. When *F_p_>C_p_*, patients tend to choose active supervision. However, when *F_p_=C_p_*, the probability of patients choosing active supervision will significantly decrease. At this time, an increasing number of patients will take the “free riding” behavior and give up supervision, but when *F_p_*<*C_p_*, the probability of patients choosing active supervision is very low, and they will finally give up supervision. Therefore, to achieve the goal of vertical integration of EHRs, the government should provide some incentives for patient supervision and try to reduce the cost of patient supervision. When the social damage *R_p_* caused by the hospital’s negative participation is greater, the patients are more inclined to adopt the active supervision strategy, but this is not what we expect (Figure 5D). Therefore, the government is required to firmly choose and strive to promote the strategy to guide and encourage hospitals to choose the active participation strategy, so as to reduce the overall social damage.

## Discussion

### Principal Findings

Using evolutionary game analysis and system dynamics simulation methods, we analyzed the evolution process of long-term strategies of the government, hospitals, and patients in the vertical integration of EHRs, and the influencing factors and mechanisms of each party’s strategy choice. The principal findings are described below.

The choice of strategies of the 3 parties is jointly determined by a variety of external factors. Increasing the benefits of the government’s efforts to promote and increasing the costs and penalties of the government’s negative treatment have a greater impact on the government’s strategic choice, while subsidies to hospitals and incentives to patients only have a little impact on the government’s strategic choice. The following scenarios would motivate hospitals to choose an active participation strategy: lower cost of active participation, higher cost of passive participation, higher government subsidies, and reasonable penalties. The revenue of hospitals under normal circumstances have a little impact on the choice of hospital’s strategies. Whether patients choose the active supervision strategy is mainly determined by the difference between the government reward and the supervision cost. Only when the patient supervision reward is higher than its cost can the purpose of encouraging patients to actively supervise be achieved.

The vertical integration of EHRs within the medical consortium has practical feasibility. In the model simulation, it is found that the strategies of the government, hospitals, and patients finally reach the optimal equilibrium state at (1, 1, 1), and both large and small hospitals have the potential willingness to take unified action to achieve the vertical integration of EHRs. However, this requires the government to be in a leading position and make every effort to promote it. It is difficult to achieve the goal of vertical integration of EHRs if the integration responsibility is fully delegated to the hospital.

Constructing a multiagent coordination mechanism under the guidance of the government is the only way to achieve the goal of vertical integration of EHRs in the medical consortium. The research shows that the government’s strategy is influenced by strategies of both hospitals and patients. Patient’s strategy is also influenced by the strategies of both the government and the hospital; the hospital’s strategy choice is mostly directly affected by the government’s strategy, and the patient’s strategy can only indirectly affect the hospital’s strategy choice through the impact on the government’s strategy. Establishing a good cooperation mechanism can not only improve the cooperation efficiency, but also reduce the participation cost, so as to accelerate the realization of the integration goal. Therefore, it is necessary to build a multibody coordination mechanism led by the government and fully mobilize the enthusiasm of all parties so that the medical consortium can achieve the goal of vertical integration of EHRs.

The simulation results revealed that the appropriate increase of government subsidies for active participation and penalties for passive participation can enhance the enthusiasm of hospitals, while the improvement of supervision incentives can also encourage patients to choose active supervision strategies. Therefore, the government should establish a reasonable reward and punishment mechanism, and design a detailed implementation plan. The government should also build a reasonable benefit distribution mechanism among medical institutions to ensure the mutual use performance after the vertical integration of EHRs. Performance evaluation plays an important role in guiding, supervising, and promoting the vertical integration, and is also the main basis for establishing a reward and punishment mechanism and a benefit distribution mechanism. Therefore, the government should independently design the performance evaluation mechanism of information resource integration in the evaluation system of medical consortium construction, to reduce “free riding” opportunistic behavior and improve the enthusiasm of hospitals and patients to participate.

### Limitations

Admittedly, there are limitations in this study. First, only the 3 core participants (the government, hospitals, and patients) were considered, while other stakeholders (eg, technology providers) were not considered. Second, because of the constraints of research conditions, real data available for prediction and simulation were not obtained. In the future, we will consider a wider range of stakeholders and seek to obtain real data for empirical studies to further improve the reliability of our findings.

### Conclusions

We should build a multiagent coordination mechanism. As discussed in the preceding sections, strategies of the government, hospitals, and patients are intertwined and each is influenced by multiple external factors. Achieving the goal of vertical integration of EHRs requires the participation of multiple parties, with the government in a leading position. Establishing a good coordination mechanism can improve the efficiency of collaboration and reduce the cost of participation, thus accelerating the achievement of integration goals. Therefore, it is imperative to establish a government-led multiparticipant collaborative mechanism.

We should set up a reward and punishment mechanism and a benefit distribution mechanism. Simulation results show that appropriately increasing government subsidies when hospitals are actively involved and penalties when they are negatively involved can increase hospital motivation. Increasing monitoring rewards can also motivate patients to choose active monitoring strategies. Therefore, the government should establish a reasonable reward and punishment mechanism and design a detailed reward and punishment implementation plan. It should also build a reasonable benefit distribution mechanism among medical institutions to ensure the interoperability performance of vertically integrated EHRs.

We should establish a scientifically integrated performance evaluation mechanism. Performance evaluation aims to compare, measure, and judge the gap between the real development status and the predefined expectation standard. The evaluation results have an important role in guiding, supervising, and promoting the development of EHRs toward verticality, and are also the main basis for setting up reward and punishment mechanisms and benefit distribution mechanisms. Therefore, the government should independently design the performance evaluation mechanism of information resource integration in the evaluation system of medical consortium construction, so as to reduce the opportunistic behavior of “free riding” and increase the enthusiasm of hospital participation.

## Abbreviations

EHR: electronic health record
